# A meta-analysis of the resuscitative effects of mechanical and manual chest compression in out-of-hospital cardiac arrest patients

**DOI:** 10.1186/s13054-019-2389-6

**Published:** 2019-03-27

**Authors:** Ni Zhu, Qi Chen, Zhixia Jiang, Futuan Liao, Bujin Kou, Hui Tang, Manhong Zhou

**Affiliations:** 1 Emergency Department, The Affiliated Hospital of Zunyi Medical University, Zunyi, 563003 China; 2 The Affiliated Hospital of Zunyi Medical University, Zunyi, China; 3 General Practice Department, The Affiliated Hospital of Zunyi Medical University, Zunyi, China

**Keywords:** Mechanical chest compression, Manual chest compression, Out-of-hospital cardiac arrest, OHCA, Meta-analysis

## Abstract

**Objectives:**

To evaluate the resuscitative effects of mechanical and manual chest compression in patients with out-of-hospital cardiac arrest (OHCA).

**Methods:**

All randomized controlled and cohort studies comparing the effects of mechanical compression and manual compression on cardiopulmonary resuscitation in OHCA patients were retrieved from the Cochrane Library, PubMed, EMBASE, and Ovid databases from the date of their establishment to January 14, 2019. The included outcomes were as follows: the return of spontaneous circulation (ROSC) rate, the rate of survival to hospital admission, the rate of survival to hospital discharge, and neurological function. After evaluating the quality of the studies and summarizing the results, RevMan5.3 software was used for the meta-analysis.

**Results:**

In total, 15 studies (9 randomized controlled trials and 6 cohort studies) were included. The results of the meta-analysis showed that there were no significant differences in the resuscitative effects of mechanical and manual chest compression in terms of the ROSC rate, the rate of survival to hospital admission and survival to hospital discharge, and neurological function in OHCA patients (ROSC: RCT: OR = 1.12, 95% CI (0.90, 1.39), *P* = 0.31; cohort study: OR = 1.08, 95% CI (0.85, 1.36), *P* = 0.54; survival to hospital admission: RCT: OR = 0.95, 95% CI (0.75, 1.20), *P* = 0.64; cohort study: OR = 0.98 95% CI (0.79, 1.20), *P* = 0.82; survival to hospital discharge: RCT: OR = 0.87, 95% CI (0.68, 1.10), *P* = 0.24; cohort study: OR = 0.78, 95% CI (0.53, 1.16), *P* = 0.22; Cerebral Performance Category (CPC) score: RCT: OR = 0.88, 95% CI (0.64, 1.20), *P* = 0.41; cohort study: OR = 0.68, 95% CI (0.34, 1.37), *P* = 0.28). When the mechanical compression group was divided into Lucas and Autopulse subgroups, the Lucas subgroup showed no difference from the manual compression group in ROSC, survival to admission, survival to discharge, and CPC scores; the Autopulse subgroup showed no difference from the manual compression subgroup in ROSC, survival to discharge, and CPC scores.

**Conclusion:**

There were no significant differences in resuscitative effects between mechanical and manual chest compression in OHCA patients. To ensure the quality of CPR, we suggest that manual chest compression be applied in the early stage of CPR for OHCA patients, while mechanical compression can be used as part of advanced life support in the late stage.

## Background

Out-of-hospital cardiac arrest (OHCA) is a sudden cardiac arrest and loss of consciousness that occurs outside of a hospital. Cardiopulmonary resuscitation (CPR) is the only effective way to rescue patients with OHCA. Chest compression is the core of modern CPR. Its quality directly affects the perfusion of the coronary and common carotid arteries and ultimately influences the prognosis of patients with cardiac arrest (CA). Some studies have reported that high-quality CPR (mainly high-quality chest compression) improves the recovery and survival rates of CA patients [1, 2]. The 2015 American Heart Association (AHA) cardiopulmonary resuscitation guidelines suggest that high-quality CPR has the following characteristics: adequate frequency, sufficient depth, full chest recoil, minimal interruptions during compressions, and avoidance of hyperventilation [3]. The advantage of manual compression as a traditional chest compression method is that it can be used to quickly intervene in the rescue of an OHCA patient without the need for any mechanical assistance; however, the compression quality is degraded by fatigue after prolonged implementation [4]. Since the first chest compression device was introduced in 1908, mechanical compression methods have been proposed and implemented in various animal studies of CPR. Because the machine does not stop or tire, it can ensure the implementation of high-quality compression with sufficient frequency, sufficient depth, and a lack of interruptions [5]. Some animal and clinical experimental studies have also shown that mechanical compression can achieve higher intrathoracic pressure than manual compression, thereby increasing blood flow and perfusion pressure in coronary and systemic circulation [6–8]. The current mechanical compression systems are mainly divided into two types. The first is a point-to-point press, which is represented by Lucas. It mainly provides compression of the lower sternum. The other type is a load-distribution press, which is represented by Autopulse. This type evenly distributes the pressure throughout the whole thorax to achieve three-dimensional pressing. The disadvantages of mechanical compression are that it requires equipment that is often not available and is not used in a timely manner. For CPR in patients with OHCA, both manual and mechanical compression have advantages and disadvantages, and there is no consensus regarding their effects and outcomes. Several recent meta-analyses have been performed, including the network meta-analysis by Khan et al. in 2018. Although that meta-analysis included 7 randomized controlled trials (RCTs), it also used network meta-analysis and showed that the 30-day survival rate, the discharge survival rate, and the nervous system function of those treated with manual compression were significantly better than those treated with the load distribution provided by Autopulse and were not significantly different from those treated with mechanical compression provided by Lucas. However, this study only performed a meta-analysis of RCTs, and our study not only added two RCTs to the original literature but also conducted a meta-analysis of cohort studies. In 2016, Li et al. conducted a meta-analysis of in-hospital and out-of-hospital CA patients; they included 8 RCTs, 3 prospective studies, and 1 descriptive study, which were combined for meta-analysis, and separately analyzed the victims of CA in the hospital and outside the hospital. Brooks et al. in 2014 and Westfall et al. in 2013 performed two meta-analyses, which included subgroup analyses despite the presence of fewer out-of-hospital RCTs and could not definitely explain the advantages and disadvantages of mechanical compression and manual compression. In 2012, Ong et al. completed only a systematic review and did not perform a meta-analysis [9–13]. Therefore, the conclusions drawn by these studies are not reliable. Hence, we conducted this meta-analysis, which includes the latest RCTs with OHCA patients and cohort studies, to investigate and compare the effects of mechanical and manual compression on the survival and prognosis of OHCA patients. To avoid data duplication, we selected only the most current study with the largest sample size when multiple studies were published by the same author. We retrieved 4 new RCTs and divided the studies into Lucas and Autopulse subgroups to further determine the advantages and disadvantages of mechanical compression and manual compression.

## Methods

### Type of study

This meta-analysis includes published randomized controlled clinical trials and cohort studies on the use of mechanical compression and manual compression to treat out-of-hospital CA. The included studies included different types of mechanical compression and manual compression groups. The patients in the studies were required to be adults with CA, and the outcome measures included the rates of the return of spontaneous circulation (ROSC), survival to hospital admission, and survival to hospital discharge, and Cerebral Performance Category (CPC) scores.

### Inclusion and exclusion criteria

#### Inclusion criteria

(1) Patients or participants: the included participants were OHCA patients. (2) Intervention measures: the comparison was between mechanical compression and manual compression. (3) Outcome indicators: the outcome indicators included primary outcome indicators, such as the rate of the ROSC, and secondary outcome indicators, such as the rate of survival to hospital admission, the rate of survival to hospital discharge, and CPC scores. Each study included at least one of the primary and one of the secondary indicators. (4) Study type: the included studies were RCTs or cohort studies.

#### Exclusion criteria

**Fig. 1:**
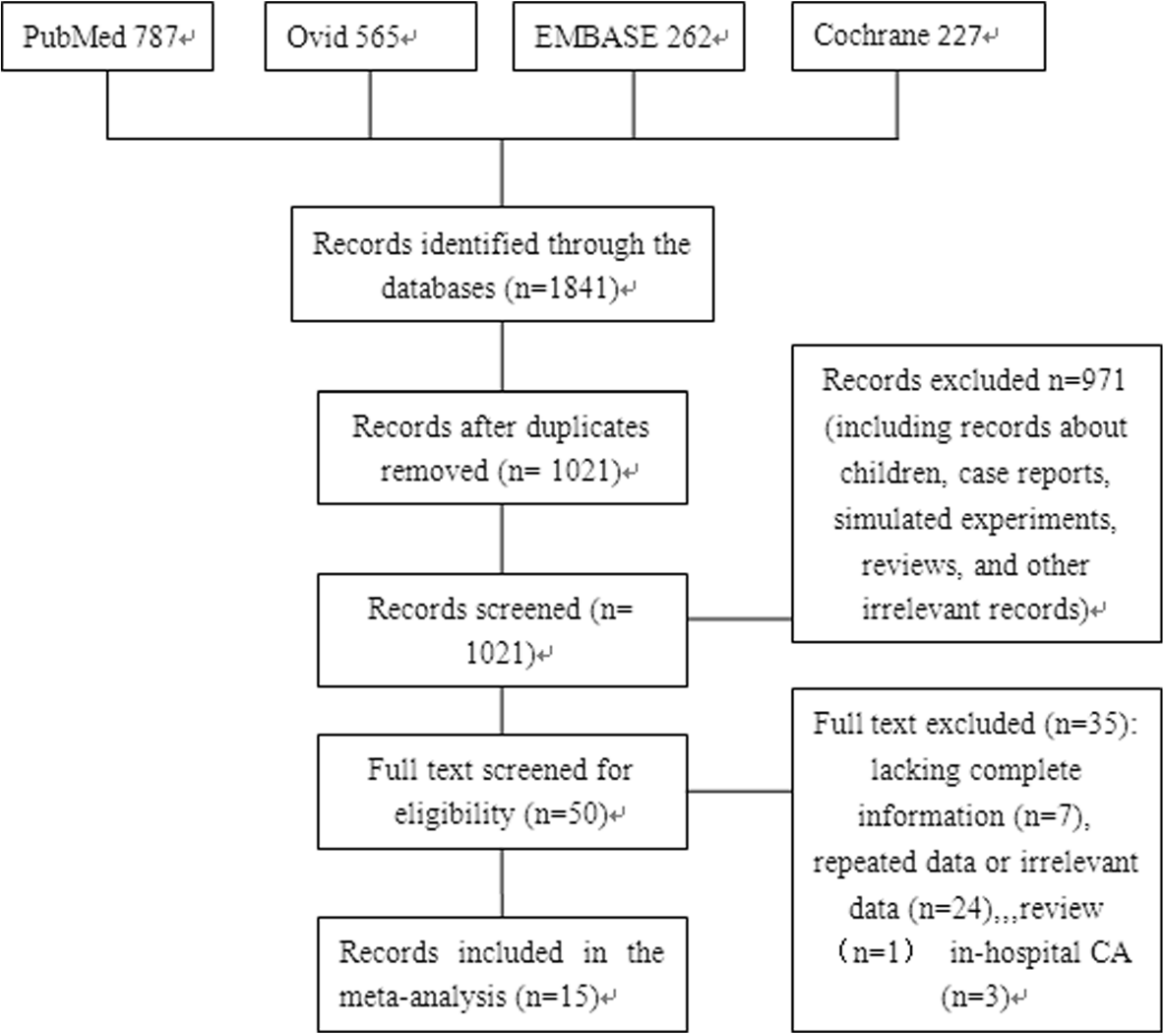
Flow diagram of the search criteria and the reasons for exclusion

The exclusion criteria were as follows: (1) the study lacked a control group or was not one of the two included types of studies. (2) The study included children younger than 18 years, animal studies, or simulation studies. (3) The original text could not be obtained, and the available information was insufficient. (4) The original data could not be transformed to be used in this study. (5) If the same institutions or individuals were found to have published a number of related studies, all but the most comprehensive study was excluded to avoid repetition. When relevant studies with different sample populations were published by the same institutions or individuals, all but the study with the largest sample size were excluded. The specific screening process is shown in Fig. 1.

### Literature search

The literature search was conducted independently by two authors. All RCTs and cohort studies comparing the effects of mechanical compression and manual compression on CPR in OHCA patients were retrieved from the Cochrane Library, PubMed, EMBASE, and Ovid databases from the date of database establishment to January 14, 2019. Keywords included “mechanical,” “manual,” “cardiopulmonary resuscitation,” and “heart arrest.”

### Data extraction

Two independent researchers collected the data from the included studies that were needed to perform a meta-analysis. If the evaluators had different opinions, they resolved the discrepancies by discussion or, if necessary, by sending the data to be evaluated by a third investigator. The outcome indicators were as follows: the primary outcome was the ROSC rate, and the secondary outcome indicators were the rate of survival to hospital admission, the rate of survival to discharge, and the neurological function score. Neurological function scores were assessed using levels 1 and 2 of the Glasgow-Pittsburgh CPC scale. The CPC score mainly revealed the patients’ neurological function at discharge.

### Literature quality evaluation

The study included RCTs and cohort studies. The quality of the included studies was independently evaluated by two authors according to the Cochrane Quality Rating Scale and the Newcastle Ottawa Scale (NOS). This study used the Cochrane scale to evaluate the RCTs; the Cochrane scale assesses the generation of random sequences, randomized concealment, blinding, and the description of outcomes. The NOS scale was used for the cohort studies; it includes the selection of the cohort, the comparability between groups, and the results and yields a possible score of 9 points.

### Data analysis

Meta-analysis of the data was performed using Review Manager 5.3, and the *χ*^2^ test and the *I*^2^ heterogeneity test were applied. *I*^2^ was used to test the heterogeneity between the included studies. Study heterogeneity was measured using the *χ*^2^ and *I*^2^ statistics, with *χ*^2^ *P* < 0.05 and *I*^2^ ≥ 50% indicating heterogeneity. According to the Cochrane systematic review mentioned in the Cochrane Handbook, as long as *I*^2^ is no more than 50%, the heterogeneity is acceptable [14]. Considering the between-study heterogeneity, a “random effects” meta-analytical technique was applied, resulting in a more conservatively calculated OR than that obtained with a fixed effects model. The count data are described as the odds ratio (OR) and its 95% confidence interval (CI). A funnel plot was used to analyze publication bias.

## Results

### Literature search

From the databases, 1841 related documents were identified; 820 articles were repeats, and 971 articles were excluded after reading the titles and abstracts. Two authors read the full texts of 50 articles, and 15 studies [15–29] were finally included in the analysis. The screening process is shown in Fig. 1.

### Document quality assessment and data extraction

The basic information for the 15 studies included the year of publication, authors’ names, country, and type of mechanical compression examined. The relevant physiological indexes and prognostic indexes included the ROSC rate, the rate of survival to hospital admission, the rate of survival to discharge, and the CPC score. Two authors read 15 articles (104,715 cases, including 78,157 in the manual compression group and 26,558 in the mechanical compression group) and summarized the quality and specific characteristics of these articles (Tables 1, 2, and 3).

**Table 1:** Cochrane quality scale for randomized controlled studies

Study	Allocation generation	Allocation concealment	Blinding of participants	Blinding of assessors	Outcome complete	Outcome selective	Other biases
1998 Edward T	High	High	Unclear	Unclear	High	Unclear	High
2006 Al Hallstrom	Medium	Medium	Unclear	Unclear	Medium	Unclear	Unclear
2011 David Smekal	Medium	Low	Unclear	Unclear	Medium	Unclear	Unclear
2014 Lars Wik	Medium	Low	Unclear	Unclear	Medium	Unclear	Unclear
2014 Sten Rubertsson	Medium	Low	Unclear	Unclear	Medium	Unclear	Unclear
2015 Gavin D Perkins	Low	Low	Unclear	Unclear	Low	Unclear	Unclear
2016 Venkataraman	Medium	Medium	Unclear	Unclear	Medium	Unclear	Unclear
2016 Chengjin Gao	Low	High	Unclear	Unclear	Medium	Unclear	Unclear
2017 Bjarne	Medium	High	Unclear	Unclear	Medium	Unclear	Unclear

**Table 2:** NOS quality scale for cohort studies

Study	Selection of cohort				Comparable	Outcomes			Score
	Selection of the exposure	Selection of the nonexposure	Determination of exposure	Initially healthy subjects	Comparable between the cohorts	Outcome measurement method	Sufficient follow-up	Integrity of follow-up	
2006 Christer Axelsson	1	1	1	1	2	1	0	0	7
2012 Marcus Eng Hock Ong	1	1	1	1	2	1	0	0	7
2015 Sebastian Zeiner	1	1	1	1	2	1	0	0	7
2015 Ching-Kuo Lin	1	1	1	1	2	1	0	0	7
2016 David G. Buckler	1	1	1	1	1	1	0	0	6
2017 Kei Hayasida	1	1	1	1	2	1	0	0	7

**Table 3:** Summary of the included studies

Year	Author	Country	Type of medical device	Setting	CPC score	ROSC	Survival to admission	Survival to discharge
					113/655:2/44	236/655:13/44	Not reported	117/655:5/44
1998	Edward T. Dickinson	USA	Thumper	Out of hospital	Not reported	Not reported	0/10:1/7	0/10:0/7
2006	Christer Axelsson	Sweden	Lucas	Out of hospital	Not reported	51/105:52/105	37/105:38/105	4/105:2/105
2006	Al Hallstrom	USA and Canada	Autopulse	Out of hospital	28/371:12/391	Not report	Not reported	37/373:23/394
2011	David Smekal	Sweden	Lucas	Out of hospital	Not reported	23/73:30/75	15/73:18/75	7/73:6/75
2012	Marcus Eng Hock Ong	Singapore	Autopulse	Out of hospital	2/459:13/552	103/459:195/552	6/459:18/552	2/459:13/552
2014	Lars Wik	Norway	Autopulse	Out of hospital	Not reported	689/2132:600/2099	Not reported	233/2132:196/2099
2014	Sten Rubertsson	Sweden, Netherlands, and UK	Lucas	Out of hospital	100/1289:108/1300	466/1289:460/1300	Not reported	118/1289:117/1300
2015	Sebastian Zeiner	Austria	Lucas and Autopulse	Out of hospital	113/655:19/239	236/655:82/239	Not reported	117/655:31/239
2015	Gavin D Perkins	UK	Lucas	Out of hospital	168/2815:77/1649	Not reported	193/2819:104/1652	Not reported
2015	Ching-Kuo Lin	Taiwan	Life-Stat 1008 Cardiopulmonary Resuscitator	Out of hospital	Not reported	51/188:72/216	33/188:48/216	Not reported
2016	David G. Buckler	USA	Not reported	Out of hospital	5976/63056:984/17625	20,493/63056:5023/17625	18,097/63056:4389/17625	7125/63056:1234/17625
2016	Chengjin Gao	China	Autopulse	Out of hospital	2/64: 5/69	15/64:31/69	Not reported	4/64:13/69
2017	Venkataraman Anantharaman	Singapore	Lucas	Out of hospital	Not reported	258/923:88/255	Not reported	27/923:13/255
2017	Kei Hayasida	Japan	Lucas and Autopulse	Out of hospital	Not reported	1561/5619:240/918	1019/5619:156/918	145/5619:23/918
2017	Bjarne Madsen Hardig	Sweden	Lucas	Out of hospital	89/383:92/374	222/383:218/374	Not reported	99:383 99:374

### Meta-analysis results

#### Return of spontaneous circulation rate

**Fig. 2:**
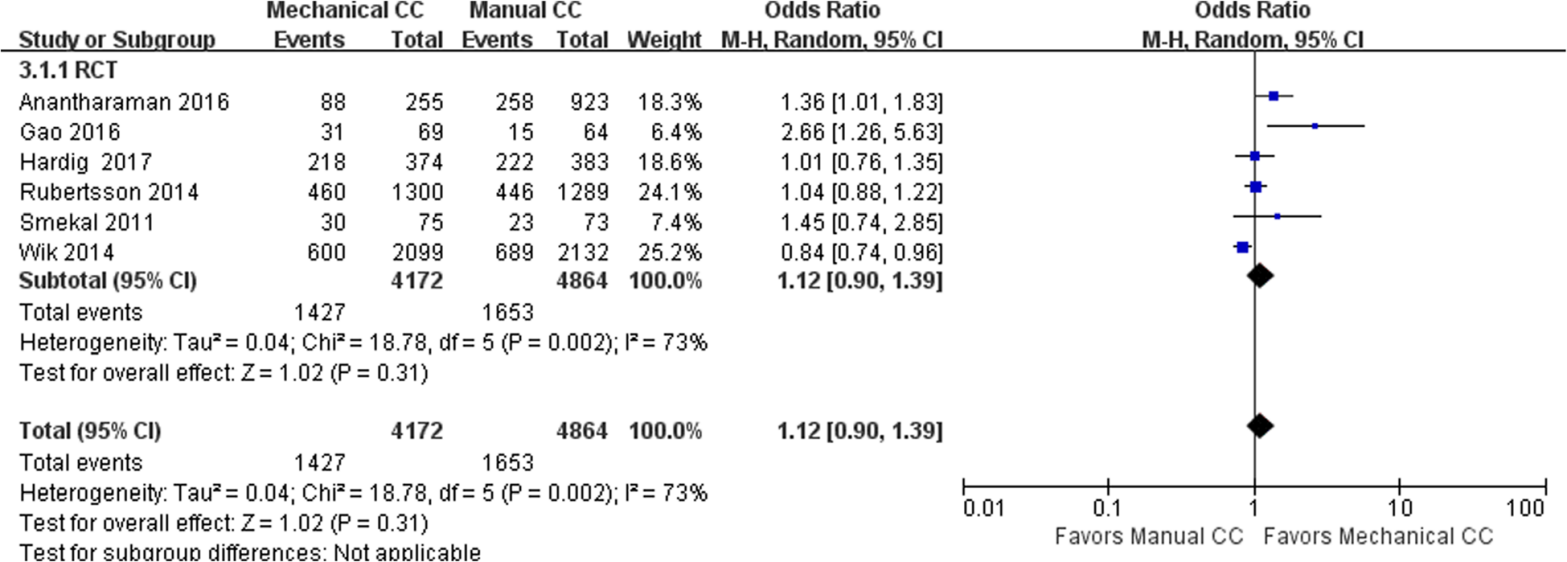
Forest plot of the ROSC for RCTs

**Fig. 3:**
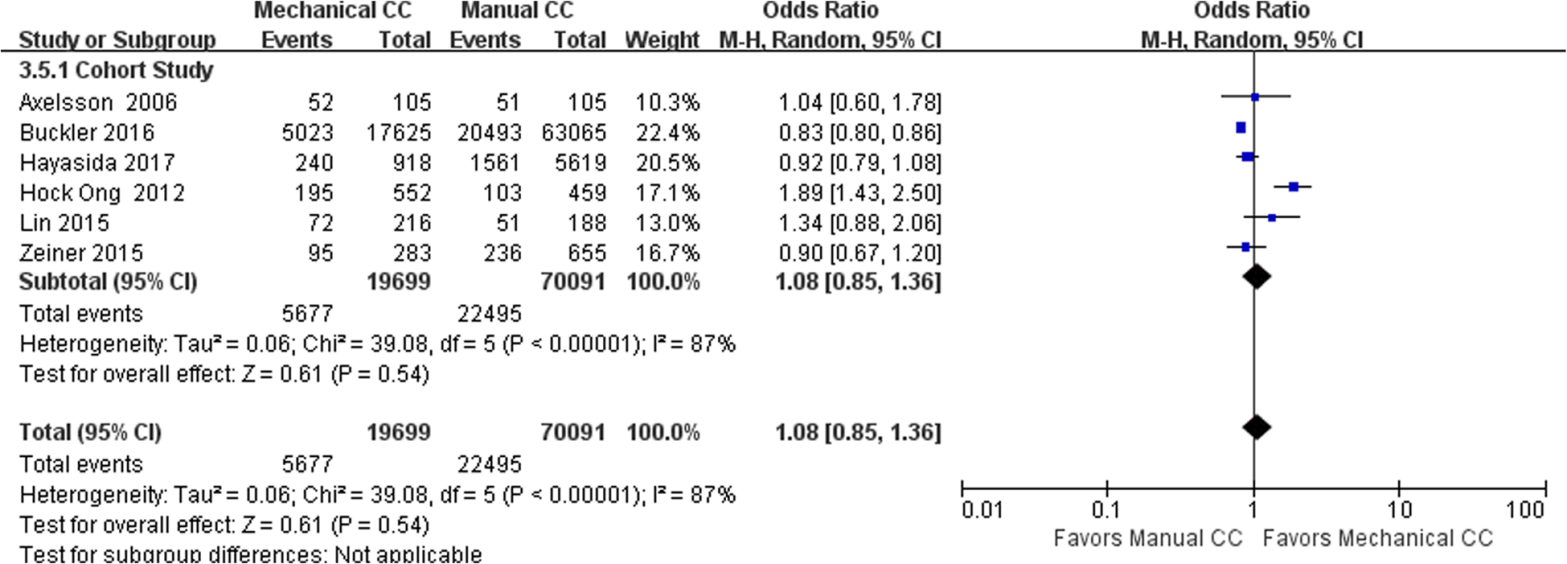
Forest plot of the ROSC for cohort studies

Twelve of the included studies (including 98,826 cases) performed statistical analyses of the observed indicators. Among these studies, there were 23,871 cases in the mechanical compression group and 74,955 cases in the manual compression group. There were 6 RCTs and 6 cohort studies. The heterogeneity test results showed that *I*^2^ was 73% and 87% for the RCTs and cohort studies, respectively, suggesting significant heterogeneity among the studies. Statistical analysis was performed using the random effects model, and no significant difference was found in the ROSC rate between the mechanical compression group and the manual compression group [RCT: OR = 1.12, 95% CI (0.90, 1.39), *P* = 0.31; cohort study: OR = 1.08, 95% CI (0.85, 1.36), *P* = 0.54]. The forest plots are shown in Figs. 2 and 3.

When the mechanical compression group was further divided into the Lucas group and the Autopulse group, the results showed that there was no heterogeneity among the 6 studies in the Lucas group (OR = 1.07, 95% CI (0.95, 1.20), *P* = 0.25). The heterogeneity among the 4 studies in the Autopulse group was *I*^2^ = 91% (OR = 1.15, 95% CI (0.94, 1.41), *P* = 0.38). There were no significant differences in the ROSC rate between the two mechanical compression subgroups and the manual compression group in patients with OHCA.

#### Survival to hospital admission

**Fig. 4:**
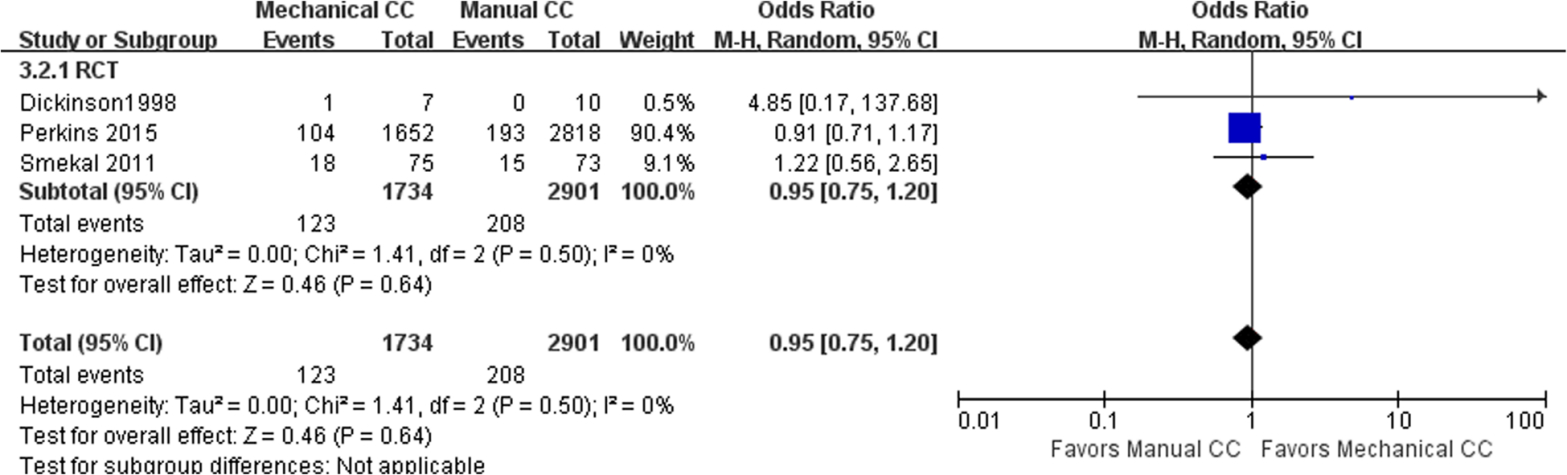
Forest plot of survival to admission for RCTs

**Fig. 5:**
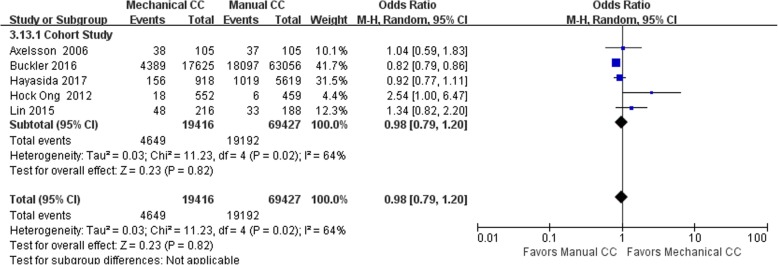
Forest plot of survival to admission for cohort studies

Eight of the included studies (including 93,478 cases) performed statistical analysis of the observed indicators. There were 72,328 cases in the manual compression group and 21,150 cases in the mechanical compression group. Among these 8 studies were 3 RCTs and 5 cohort studies. The heterogeneity test results showed that *I*^2^ was 0% and 64% for the RCTs and cohort studies, respectively, indicating there was no heterogeneity in the RCTs and significant heterogeneity in the cohort studies. The statistical analysis was performed using the random effects model, which showed no significant difference in the rate of survival to hospital admission between the mechanical compression group and the manual compression group [RCT: OR = 0.95, 95% CI (0.75, 1.20), *P* = 0.64; cohort study: OR = 0.98 95% CI (0.79, 1.20), *P* = 0.82]. The forest plots are shown in Figs. 4 and 5. When the mechanical compression group was further divided into the Lucas group and the Autopulse group, the results showed that there was no heterogeneity among the 3 studies in the Lucas group and no difference between the Lucas subgroup and the manual compression group (OR = 0.95, 95% CI (0.77, 1.81), *P* = 0.66). In the Autopulse subgroup, only one result published by Marcus Eng Hock Ong in 2012 showed that the survival rate of Autopulse admission was superior to that of the manual compression group; hence, more research is needed to further validate this result.

#### Survival to hospital discharge

**Fig. 6:**
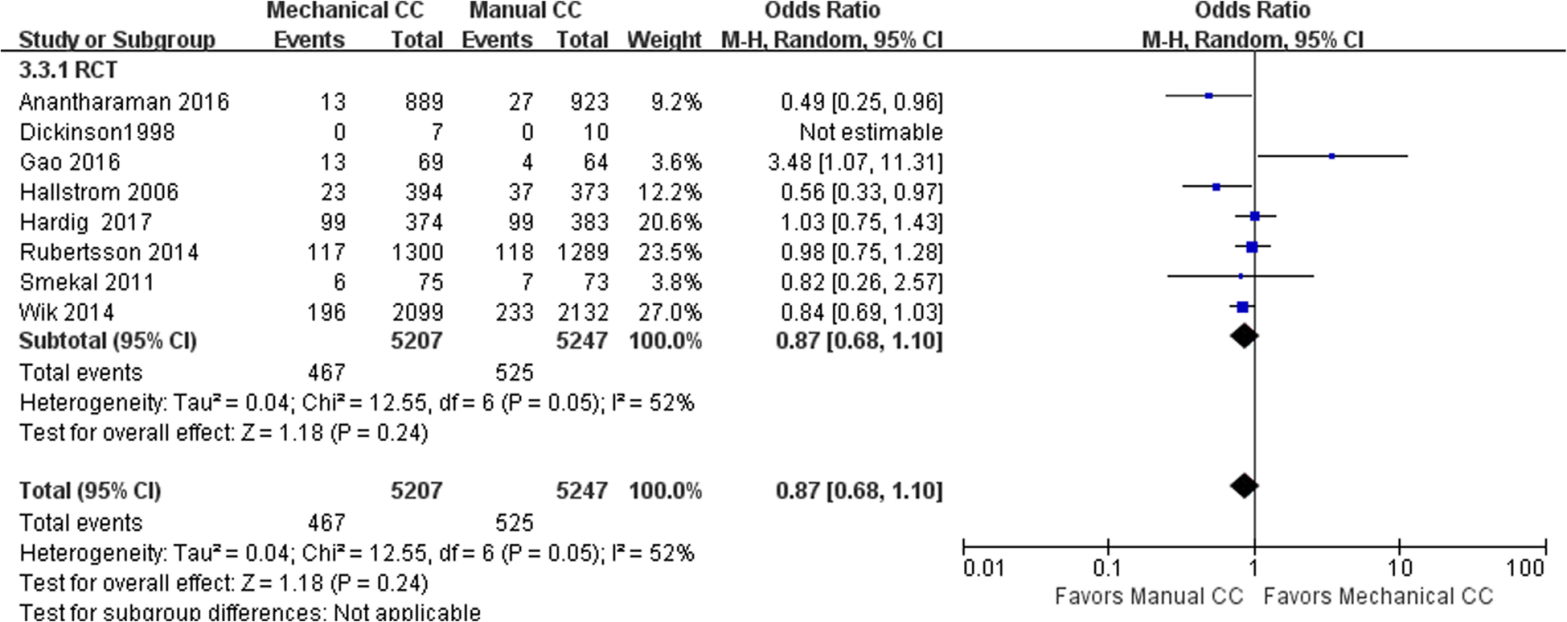
Forest plot of survival to discharge for RCTs

**Fig. 7:**
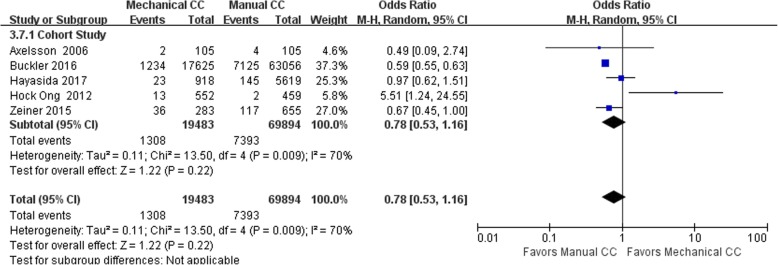
Forest plot of survival to discharge for cohort studies

Thirteen of the included studies (including 99,831 cases) performed a statistical analysis of the observed indicators. Among them, there were 75,141 cases in the manual compression group and 24,690 cases in the mechanical compression group. The heterogeneity results for RCTs and cohort studies showed that *I*^2^ was 52% and 70%, respectively, suggesting that there was heterogeneity among the studies. Hence, the random effects model was applied for the statistical analysis of the indicators; the analysis showed that there was no significant difference in the rate of survival to hospital admission between the manual compression group and the mechanical compression group [RCT: OR = 0.87, 95% CI (0.68, 1.10), *P* = 0.24; cohort study: OR = 0.78, 95% CI (0.53, 1.16), *P* = 0.22]. The forest plots are shown in Figs. 6 and 7.

When the mechanical compression group was further divided into the Lucas group and the Autopulse group, the results showed that there was no heterogeneity among the 6 studies in the Lucas group, *I*^2^ = 20% (OR = 0.86, 95% CI (0.69, 1.06), *P* = 0.16). The heterogeneity among the 5 studies in the Autopulse group was *I*^2^ = 72% (OR = 1.06, 95% CI (0.59, 1.91), *P* = 0.84). The random effects model was applied for the statistical analysis of the indicators. The final results showed no significant difference in the rate of survival to discharge between the two subgroups of mechanical compression and the manual compression group in patients with OHCA.

#### CPC score

**Fig. 8:**
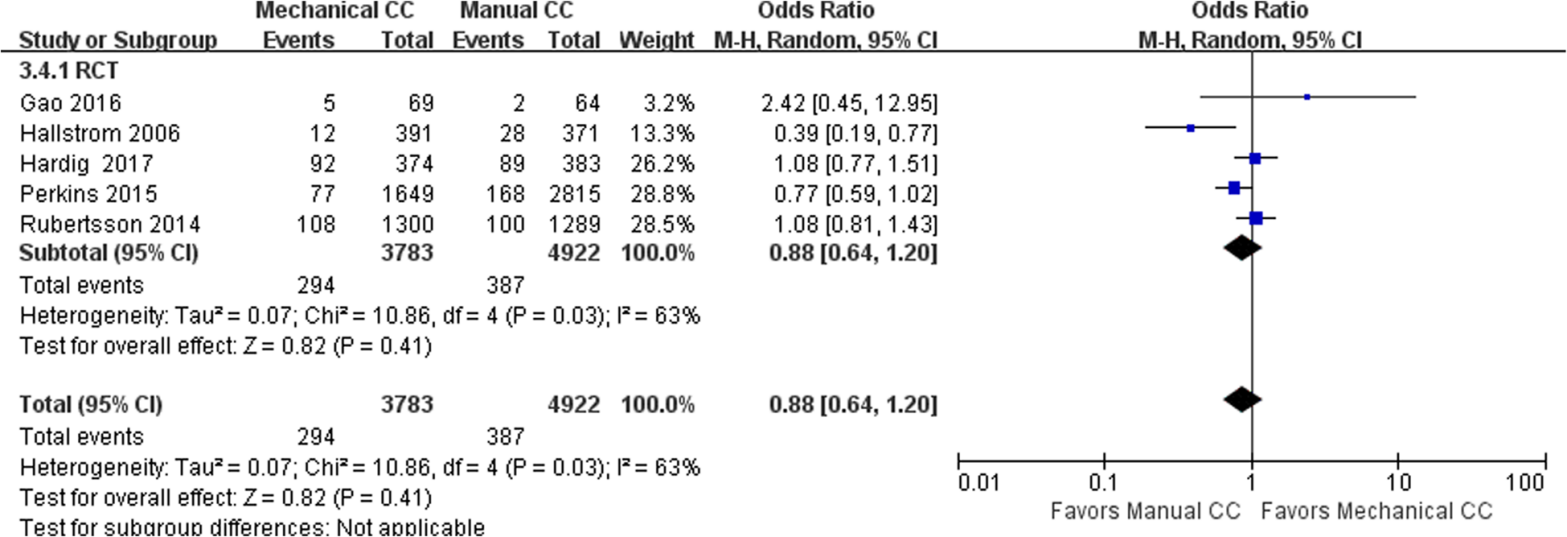
Forest plot of CPC scores for RCTs

**Fig. 9:**
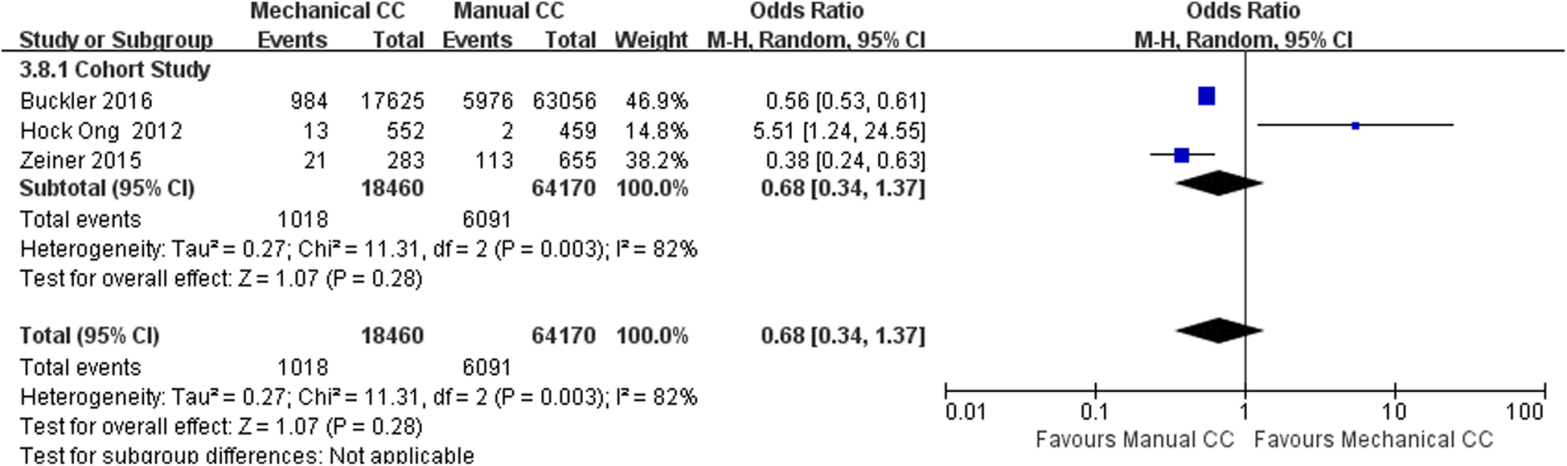
Forest plot of CPC scores for cohort studies

Eight of the included studies (including 91,335 cases) performed statistical analysis of the observed indicators. Among them, there were 69,092 cases in the manual compression group and 22,243 cases in the mechanical compression group. The heterogeneity test results showed that *I*^2^ was 63% for the RCTs and 82% for the cohort studies, suggesting that the heterogeneity among the studies was high. Therefore, the random effects model was used to analyze the observed indicators. No significant difference in the CPC score was found between the manual compression and mechanical compression groups [RCT: OR = 0.88, 95% CI (0.64, 1.20), *P* = 0.41; cohort study: OR = 0.68, 95% CI (0.34, 1.37), *P* = 0.28]. The forest plots are shown in Figs. 8 and 9.

Subgroup analysis showed higher heterogeneity among the studies in both the Lucas group and the Autopulse group, with *I*^2^ = 76% and *I*^2^ = 79%, respectively. Therefore, the random effects model was applied for the statistical analysis, and it showed no significant difference in the CPC scores of the manual compression group and the mechanical compression group. The forest plot is shown in Fig. 8. One explanation for the lack of an observed difference is the fact that the CPC score is relatively subjective, even though there are consistent standards; consequently, the differences between the studies are relatively large.

### Study publication bias

The funnel plot that analyzed the rate of ROSC, the rate of survival to hospital admission, the rate of survival to discharge, and the CPC score in the OHCA patients in both the mechanical compression group and the manual compression group showed that most of the studies in the figure are symmetrical and adhere to the inverted funnel shape, which indicates that the publication bias is low.

## Discussion

The application of CPR for CA patients aims to restore spontaneous circulation as soon as possible and to obtain better neurological function after discharge. The 2015 AHA guidelines for CPR emphasize the importance of high-quality CPR for the survival of CA patients [3]. Current methods of compression include manual compression and mechanical compression, each of which has advantages and disadvantages. The network meta-analysis by Khan et al. [13] in 2018 showed that manual compression was significantly better than mechanical compression in 30-day survival, discharge survival, and neurological function, especially over load-distributed distribution press provided by Autopulse; however, it had no significant difference with the Lucas mechanical press. Moreover, manual compression was better than the Autopulse mechanical compression in the formation of pneumothorax and hematoma. Li et al. [12] performed a meta-analysis of in-hospital and out-of-hospital CA patients in 2016 and showed that for out-of-hospital CA, there was no significant difference in CPC score, admission survival rate, and discharge survival rate between manual compression and mechanical compression, and that manual compression was superior to mechanical compression in the rate of spontaneous circulation recovery, especially superior to mechanical compression provided by Autopulse. For in-hospital CA, manual compression was superior to mechanical compression in the rate of spontaneous circulation recovery and discharge survival [12]. Brooks et al. [11] conducted a meta-analysis in 2014, only analyzed the neurological function scores, and showed that manual compression was superior to mechanical compression. Westfall et al. [9] conducted a meta-analysis in 2013 and showed that the mechanical compression provided by LBD was superior to manual compression in the rate of spontaneous circulation recovery, but there was no difference in the rate of spontaneous circulation recovery between the piston-driven cardiopulmonary resuscitation and manual compression. There were no consistent results in the previous results about the superiority of mechanical compression and manual compression, so the conclusions may be not reliable. This meta-analysis included 15 relevant studies: 9 RCTs and 6 cohort studies. The results showed that there were no differences between manual compression and mechanical compression in terms of the four observed indicators (the ROSC rate, the rate of survival to hospital admission, the rate of survival to hospital discharge, and CPC scores). In addition to the ROSC rate, the results of the remaining three outcome measures were consistent with the results of the meta-analysis of outpatients with CA performed by Li et al. in 2016 [12]. When mechanical compression was divided into Lucas and Autopulse subgroups, the results showed no significant difference between the Lucas subgroup and the manual compression group for the ROSC, survival to admission rate, survival to discharge rate, and CPC scores. There were no differences between the Autopulse subgroups and the manual compression group for the ROSC, survival to discharge rate, and CPC score. The Autopulse subgroup was superior to the manual compression group in terms of the survival to admission rate, but this conclusion was drawn from only one study (Marcus Eng Hock Ong’s study in 2012), and the study uses manual compression to reduce the duration of compression interruption before and Autopulse application. Therefore, the study may have a large bias, and more original studies are needed to further verify the results. The possible reasons for the above results are as follows: (1) Although most of the studies included were RCTs, the descriptions of the randomization methods, such as the generation of random sequences, were not specific; additionally, there were significant differences in the way in which compression was performed, and the requirements for blinding were not met. Some studies [21–23, 25, 26] used two compression methods; however, mechanical compression requires more preparation time than manual compression, and CPR requires early chest compression. Due to the need to save the patient’s life, both the experimental group and the control group first received manual compression and then were grouped into the manual and mechanical compression groups. (2) In actual situations, mechanical compression can be often used for patients in whom early defibrillation cannot be applied or for CA patients who require long-term chest compressions. These CA patients usually have a worse prognosis than patients who receive early defibrillation or who experience early ROSC. Because most of the studies included stratification of shockable and nonshockable rhythm and CA with witnesses and CA without witnesses, further meta-analysis was performed between patients with shockable and nonshockable rhythm and patients with witnessed CA and unwitnessed CA. For nonshockable CA and shockable CA, the included studies showed no significant difference between the mechanical compression group and the manual compression group (nonshockable CA: OR = 1.10, 95% CI (0.93, 1.31), *P* = 0.26; shockable CA: OR = 0.87, 95% CI (0.72, 1.06), *P* = 0.16); there was also no significant differences between the witness and unwitnessed CA subgroups (unwitnessed CA: OR = 0.94, 95% CI (0.71, 1.25), *P* = 0.68; witnessed CA: OR = 1.07, 95% CI (0.81, 1.41), *P* = 0.66); however, there was a lack of detailed descriptions of the populations that received mechanical compression and manual compression, e.g., whether the CA victims had unpredictable factors such as ineffective early defibrillation and a need for long-term compression. It is impossible to determine whether there was selection bias in the included studies. (3) Complications or comorbidities of CPR have significant impacts on the prognosis of CA patients. Although some studies have shown that the type and extent of injury caused by manual compression is not significantly different from that caused by mechanical compression [30], CA patients who receive mechanical compression are more likely to develop rib or sternal fractures, pneumothorax and subcutaneous emphysema [23], resulting in more complex treatment after admission and an increased risk of death. Therefore, it seems that mechanical compression is not better than manual compression. The network meta-analysis by Khan et al. in 2018 also showed that compared with mechanical compression, manual compression led to less pneumothorax and hematoma [13]. This finding requires further meta-analysis or RCTs to verify. (4) At the same time, the current mechanical compression systems have drawbacks. Although the mechanisms of cardiac compression can be simulated well, mechanical compression systems, from the early Thumper system to the current Lucas and Autopulse systems, have consistently lacked quality detection methods and cannot indicate the quality and safety of chest compressions in a timely manner. These systems are not suitable for very large or very small patients, and they are difficult to transport, rendering them less useful for the early application of compression. In fact, in addition to the effects of timely and uninterrupted high-quality compression on the prognosis of CA patients, other factors, such as the time between cardiac arrest and treatment and the type of initial arrhythmia, also affect the quality of CPR. As described in a prospective, multicenter, observational article by Hayashida in 2017, mechanical compression may be associated with a poor prognosis for CA victims given the potential confounders of out-of-hospital first aid. It is believed that further research is needed to clarify whether there is a benefit to mechanical compression [29]. Therefore, it can be concluded that mechanical chest compression is not superior to manual chest compression.

The limitation of this meta-analysis lies in the fact that it is a secondary analysis of original literature. Although most of the original studies included were RCTs, their quality scores were low, and the included studies did not include Chinese patients; the majority of the medical records examined in each study were from Europe and the USA. This may lead to a geographical limitation on the applicability of the results. This meta-analysis targeted adult OHCA patients but included different age groups. In addition, different individuals may have different underlying diseases, and the causes of OHCA in different individuals may affect the quality of CPR. At the same time, the results of the study also suggest that regardless of how technology develops, manual compression is the most timely method of CPR, and it is an effective way to rescue patients with OHCA.

## Conclusion

This meta-analysis included 15 relevant studies. There were no significant differences in the rate of ROSC, rate of survival to admission, rate of survival to discharge, and neurological function scores between the manual compression and mechanical compression groups. When the mechanical compression group was divided into Lucas and Autopulse subgroups, the results showed that the Lucas subgroup did not differ significantly in the ROSC, admission survival rate, discharge survival rate, and CPC scores compared with the manual compression group. The Autopulse subgroup had no difference in ROSC, discharge survival rate, and CPC scores compared with the manual compression group. However, manual compression is more advantageous because it can be implemented in a timely and effective manner, while mechanical compression tends to cause more complications. Therefore, we recommend that manual compression be performed in the early stage of CPR to rescue OHCA patients. Mechanical compression can be used as part of advanced life support, but it cannot currently completely replace timely manual compression.
